# The Impact of Comorbidities on Calgary Hospital Utilization in Patients With Chronic Obstructive Pulmonary Disease and Heart Failure

**DOI:** 10.7759/cureus.17303

**Published:** 2021-08-19

**Authors:** Douglas C Woodhouse, Alexandra D Frolkis, Brenna J Murray, Nathan M Solbak, Najla Samardzic, Kelly W Burak

**Affiliations:** 1 Family Medicine, University of Calgary, Calgary, CAN; 2 Internal Medicine, University of Calgary, Calgary, CAN; 3 Medicine, University of Calgary, Calgary, CAN

**Keywords:** copd, heart failure, length of hospital stay (los), hospitalization, medical comorbidities

## Abstract

Background

Chronic obstructive pulmonary disease (COPD) and heart failure (HF) are chronic conditions with high acute care utilization. Disease-specific order sets were developed for patients with COPD or HF in Calgary to reduce total days in hospital for this population of patients. However, many patients have comorbidities which may contribute to hospital utilization; thus, disease-specific order sets may not be an optimal solution to reduce overall acute care utilization.

Methods

Inpatient data on Calgary hospitalizations for COPD or HF between April 1, 2017 - March 31, 2019 and associated diagnoses were identified. Outcomes included total days in hospital and length of stay for COPD and HF patients stratified by number of comorbidities.

Results

Total days in hospital increased with the number of comorbidities for both conditions. During the study period, 131 patients with COPD and no comorbidities had a median length of stay of three days (IQR: 3) compared to 3,911 COPD patients with one to five comorbidities with a median length of stay of seven days (IQR: 9). There were 47 patients with HF and no comorbidities with a median length of stay of four days (IQR: 5) compared to 6,273 HF patients with one to five comorbidities with a median length of stay of nine days (IQR: 12). Common comorbidities included hypertension, type 2 diabetes, and acute renal failure. COPD and HF are frequently comorbid.

Conclusions

Total days in hospital for patients with COPD or HF is positively correlated with the number of comorbidities. COPD or HF patients with between one to five comorbidities (compared to those with no comorbidities, and those with more than five comorbidities) represent the majority of total days in hospital, and the majority of patients. This highlights the importance of focusing on patients with comorbidities in efforts to reduce hospital utilization, and suggests that concurrent management of commonly occurring comorbidities for HF and COPD patients may be necessary to achieve this goal.

## Introduction

Chronic obstructive pulmonary disease (COPD) and heart failure (HF) are chronic conditions with high morbidity and acute care utilization. They are among the top four diagnoses for inpatient hospitalizations in Alberta [1]. Many guidelines have been developed to encourage wider adoption of evidence-based practices for the management of patients with these conditions [2,3]. An estimated $1 billion was spent from 2008 to 2016 in Alberta for COPD care, with the majority of costs associated with hospitalization [4]. Evidence-based, disease-specific acute care order sets for COPD and HF are being implemented in Calgary hospitals, with an intention to spread provincially and to community care [5]. The primary goal is to reduce total days in hospital for patients with either of these conditions. However, the order sets are not designed to optimize the coordinated management of commonly occurring co-morbidities, potentially limiting their clinical utility.

At least one comorbidity (hypertension, acute myocardial infarction, atrial fibrillation, HF, and diabetes mellitus) has been observed in >95% of patients with COPD [6]. Management of COPD is challenging due to the prevalence of comorbidities such as HF and risk factors such as diabetes or hypertension [7]. Comorbidities are associated with COPD exacerbation risk and increased length of stay [8].

The prevalence of comorbidities in HF is also high, with up to 40% of patients having five or more non-cardiac comorbidities [9]. The proportion of HF patients with five or more chronic comorbid conditions is increasing over time, from 42% (between 1988-1994) to 58% (between 2003-2008) [10]. Similarly to COPD, comorbidities are associated with higher likelihood of readmission [11] and a significantly longer mean length of stay [12].

Our objective was to evaluate the frequency of comorbidities among patients with COPD or HF admitted to Calgary hospitals and their influence on length of stay and total patient-days in hospital. A secondary objective was to identify the most commonly occurring co-morbidities with COPD and HF.

## Materials and methods

Data sources

The province of Alberta in Canada publicly administers healthcare to all residents and tracks access to healthcare using a unique personalized healthcare number (PHN). All hospital discharges for individuals with a valid PHN are recorded and compiled in the Inpatient Discharge Abstract Database. This dataset includes up to 25 diagnostic codes using the International Classification of Disease 10th version (ICD-10) and provides information on hospital location as well as admission and discharge date which, allows for calculation of length of hospital stay.

Study population

The Discharge Abstract Database was used to identify all patients over the age of 18 with a valid PHN admitted to a Calgary hospital from April 1, 2017 to March 31, 2019 who had an ICD-10 diagnostic code for either COPD or HF recorded in any position in their discharge abstract. COPD was defined with ICD-10 codes J41, J42, J43, and J44 which have been validated in population-based health administrative databases and compared to chart reviews for sensitivity [13]. HF was defined with ICD-10 code I50 which has been validated with administrative data [14]. Individuals who died during the study period were included.

Case ascertainment

For our analysis, we created a ‘patient profile’ by assigning all diagnosis codes associated with a patient for any admission during the study period to that patient for all admissions during the study period, even if it was not coded as a diagnosis on a particular hospital admission. The rationale for a ‘patient profile’ is that any recently documented diagnosis is likely to have contributed to overall patient morbidity, complexity of management, and total days in hospital or readmission during the study period [15]. The primary outcome was length of hospital stay in days. The cumulative number of days in hospital over the study period for each patient profile was calculated.

Age at initial admission over the study period, sex, and comorbidities were explored as covariates. Comorbidities were defined using the Elixhauser Comorbidity Index and analyzed both as a continuous variable, and stratified as: 1) 0 comorbidities (a single diagnosis of either COPD or HF); 2) between one and five comorbidities inclusive; and 3) greater than five comorbidities. Coding algorithms to define the 31 Elixhauser comorbidity groupings using ICD-10 codes have been previously reported by Quan et al. [16]. Five comorbidities was chosen because several studies have found this to be the mean number of comorbidities among patients admitted with COPD and HF [17,18]. 

Statistical analysis

Analyses were performed separately for COPD and HF. Baseline characteristics were explored descriptively. Proportions were described as percentages, and continuous variables were described as means with standard deviations (SD), and medians with first and third quartiles (Q1, Q3). Wilcoxon rank-sum tests and Kruskal-Wallis non-parametric tests were used to compare continuous variables, and chi-squared tests were used to compare proportions. Crude and adjusted (for age and sex) multilevel linear regression was performed with person as the hierarchy to explore how number of Elixhauser comorbidities as a continuous variable over the study period correlated with length of stay with results reported as coefficients and 95% confidence intervals (CI). STATA/SE 14.2 (StataCorp, Texas, USA) and Oracle SQL were used for all statistical analyses with an alpha of 0.05. Graphics were performed using Tableau and Microsoft Excel. Ethics approval was granted through the Conjoint Health Research Ethics Board at the University of Calgary (Reference number: REB19-1520).

## Results

Between April 1, 2017 and March 31, 2019, the total number of distinct adult patients with COPD or HF was 5,032 and 7,197 respectively; 131 patients with COPD had no comorbidities, 3,911 had one to five comorbidities, and 990 had more than five comorbidities. 47 patients with HF had no comorbidities, 6,273 had one to five comorbidities, and 877 had more than five comorbidities. Baseline characteristics of the study cohort are presented in Table 1.

**Table 1:** Demographic and Elixhauser comorbidity data of patients with COPD or heart failure a - Elixhauser comorbidities defined by ICD-10 are reported by Quan et al. 2005 [16] b - Mortality during study period COPD - chronic obstructive pulmonary disease, Q1 - first quartile, Q3 - third quartile

Category	COPD (n=5,032)	Heart failure (n=7,197)
Age in years, median (Q1, Q3)	74 (65, 82)	79 (68, 86)
Sex, (n, %)		
Female	2,436 (48.4)	3,526 (48.9)
Male	2,596 (51.6)	3,671 (51.1)
Number of visits, median (Q1, Q3)	1 (1, 2)	1 (1, 2)
Length of stay in days, median (Q1, Q3)	10 (5, 23.5)	14 (7, 30)
Elixhauser comorbidities, (n, %)^a^		
Congestive heart failure	1,427 (28.4)	7,197 (100.0)
Cardiac arrhythmias	1,173 (23.3)	3,258 (45.3)
Valvular disease	191 (3.8)	934 (13.0)
Peripheral vascular disorder	160 (3.2)	243 (3.4)
Hypertension – uncomplicated	1,741 (34.6)	3,305 (45.9)
Hypertension – complicated	40 (0.8)	206 (2.9)
Chronic pulmonary disease	5,032 (100.0)	1,463 (20.3)
Pulmonary circulation disorder	345 (6.9)	579 (8.1)
Diabetes – uncomplicated	337 (6.7)	102 (1.4)
Diabetes – complicated	1,014 (20.2)	2,679 (37.2)
Hypothyroidism	61 (1.2)	136 (1.9)
Fluid and electrolyte disorders	1,082 (21.5)	1,914 (26.6)
Renal failure	374 (7.4)	958 (13.3)
Lymphoma	51 (1.0)	78 (1.1)
Metastatic cancer	233 (4.4)	172 (2.4)
Solid tumor without metastasis	447 (8.9)	331 (4.6)
Paralysis	39 (0.8)	67 (0.9)
Other neurologic disorders	115 (2.3)	190 (2.6)
Liver disease	157 (3.1)	247 (3.4)
Peptic ulcer disease excluding bleeding	20 (0.4)	29 (0.4)
Alcohol misuse	305 (6.1)	183 (2.5)
Drug misuse	111 (2.2)	76 (1.1)
Psychoses	47 (0.9)	35 (0.5)
Depression	159 (3.2)	232 (3.2)
Deficiency anemia	200 (4.0)	403 (5.6)
Blood loss anemia	38 (0.8)	108 (1.5)
AIDS/HIV	4 (0.1)	1 (0.0)
Rheumatoid arthritis/collagen vascular disorder	71 (1.4)	96 (1.3)
Coagulopathy	319 (6.3)	716 (10.0)
Obesity	138 (3.8)	260 (3.6)
Weight loss	197 (3.9)	218 (3.0)
Mortality, (n, %)^b^	197 (3.9)	395 (8.2)

COPD

The mean (SD) number of comorbidities was 2.7 (1.3) and 6.8 (1.1) for patients with one to five and greater than five comorbidities, respectively. The most common comorbidities for COPD patients were uncomplicated hypertension (34.6%), congestive HF (28.4%), arrhythmias (23.3%), fluid and electrolyte disorders (21.5%), and complicated diabetes (20.2%) (Table 1).

**Figure 1:**
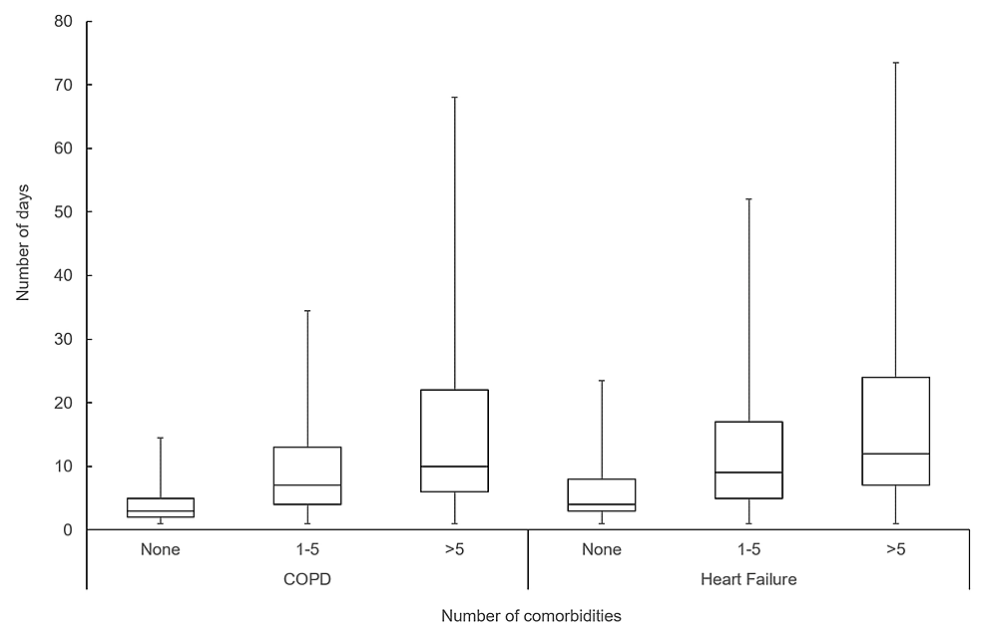
Median length of stay stratified by the number of Elixhauser comorbidities for patients with COPD and heart failure COPD - chronic obstructive pulmonary disease

There was a significant difference in age, number of visits, and length of stay based on number of Elixhauser comorbidities (Table 2). Patients with no comorbidities (a single diagnosis of COPD) had significantly lower median acute length of stay (median 3; IQR: 3) compared to those with one to five comorbidities (median 7; IQR: 7) and more than five comorbidities (median 10; IQR: 14) with p-value <0.001 (Figure 1). The number of comorbidities was significantly associated with increased length of hospital stay both as a crude estimate (coefficient 4.9; 95% CI 4.4-5.5; p<0.001) and after adjusting for age and sex (coefficient 4.9; 95% CI 4.4-5.4; p<0.001) (Table 2).

**Table 2:** Differences between stratification of diagnoses in characteristics and outcomes based on the number of Elixhauser comorbidities in COPD patients a - No comorbidities during any hospital visit over the two year study period b – Length of stay in hospital per admission c – Total cumulative days in hospital for all patients over the two year study period COPD - chronic obstructive pulmonary disease, Q1 - first quartile, Q3 - third quartile

Category	No comorbidities^a ^(n=131)	One to five comorbidities (n=4,436)	More than five comorbidities (n=465)	p-value
Sex, n (%)				0.28
Female	68 (51.9)	2,161 (48.7)	207 (44.5)	
Male	63 (48.1)	2,275 (51.3)	258 (55.5)	
Age, median (Q1, Q3)	68 (61, 77)	74 (65, 82)	74 (67, 82)	<0.001
Number of visits, median (Q1, Q3)	1 (1, 1)	1 (1, 2)	2 (1, 4)	<0.001
Length of stay, median (Q1, Q3)^b^	3 (2, 5)	7 (4, 13)	10 (6, 22)	<0.001
Cumulative days in hospital^c^	490	62,504	12,166	<0.001
Mortality, n (%)	0 (0.0)	176 (4.0)	21 (4.5)	0.055

**Figure 2:**
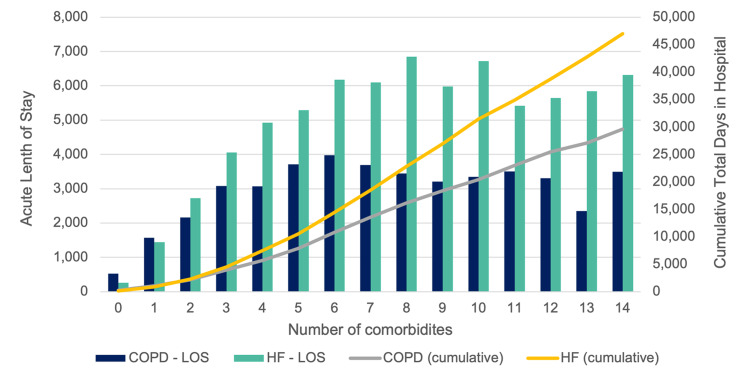
Total days in hospital and cumulative total days in hospital for all patients with COPD or HF (including acute and alternate level of care days in hospital over the two-year study period) COPD - chronic obstructive pulmonary disease, HF - heart failure, LOS - length of stay

The cumulative number of days in hospital during the study period (including acute and ALC days for all patients) was 525 for those with no comorbidities, 88,855 for those with 1-5 comorbidities, and 26,167 for those with more than five comorbidities (Figure 2). 

HF

There was a mean (SD) number of 3.2 (1.2) and 6.6 (1.0) comorbidities in patients with one to five and greater than five comorbidities, respectively. HF patients’ most common comorbidities were uncomplicated hypertension (45.9%), arrhythmias (45.3%), complicated diabetes (37.2%), fluid and electrolyte disorders (26.6%) and chronic pulmonary disease (20.3%) (Table 1).

There was a significant difference in median length of stay for patients based on number of Elixhauser comorbidities. Patients with no comorbidities (a single diagnoses of HF) had a significantly shorter length of stay (median 4; IQR: 5) compared to those with one to five comorbidities (median 9; IQR: 12); and those with more than five comorbidities (median 12; IQR: 21) (p-value <0.001) (Figure 1). The number of comorbidities was significantly associated with increased length of hospital stay both as a crude estimate (coefficient 3.9; 95% CI 3.5-4.2; p<0.001) and after adjusting for age and sex (coefficient 4.0; 95% CI 3.6-4.3; p<0.001) (Table 3).

**Table 3:** Differences between stratification of diagnoses in characteristics and outcomes based on the number of Elixhauser comorbidities in heart failure patients a - No comorbidities during any hospital visit over the two-year study period b – Length of stay in hospital per admission c – Total cumulative days in hospital for all patients over the two-year study period Q1 - first quartile, Q3 - third quartile

Category	No comorbidities^a ^(n=47)	One to five comorbidities (n=6,273)	More than five comorbidities (n=877)	p-value
Sex, n (%)				0.205
Female	23 (48.9)	3,098 (49.4)	405 (46.2)	
Male	24 (51.1)	3,175 (50.6)	472 (53.8)	
Age, median (Q1, Q3)	77 (67, 87)	79 (68, 87)	75 (67, 83)	<0.001
Number of visits, median (Q1, Q3)	1 (1, 1)	1 (1, 2)	2 (1, 3)	<0.001
Length of stay, median (Q1, Q3)^b^	4 (3, 8)	9 (5, 17)	12 (7, 28)	<0.001
Cumulative days in hospital^c^	262	100,220	22,548	<0.001
Mortality, n (%)	0 (0.0)	351 (5.6)	44 (5.0)	0.198

The cumulative number of days in hospital during the study period (including acute and ALC days for all patients) was 262 for those with no comorbidities, 135,555 for those with one to five comorbidities, and 47,247 for those with more than five comorbidities (Figure 2).

## Discussion

In this retrospective study, we sought to determine the impact of number of comorbidities on total patient-days in hospital for patients with COPD and HF. The majority of patient-days in hospital were attributable to patients with one to five comorbidities (despite a shorter median length of stay when compared to those with more than five comorbidities). This suggests that efforts to reduce patient-days in hospital could focus on patients with one to five comorbidities, who represent ‘average’ patients, compared to ‘outliers’ who have either a single diagnosis (for whom a disease-specific order set is most appropriate) or more than five comorbidities (for whom a disease-specific order set is least appropriate). The most common co-morbidities associated with COPD and HF, including uncomplicated hypertension, arrhythmias, complicated diabetes and fluid, and electrolyte imbalances.

In the first Canadian national study to identify comorbidities with HF, hypertension, diabetes, and acute renal failure were commonly diagnosed [19]. International studies in Italy [6] and the United States [8] support the association between cardiovascular comorbidities in COPD patients and extended hospitalization. Average acute LOS at the four Calgary hospitals were comparable with national averages [1]. The similarities with our results suggest that our findings and suggestions are likely generalizable to other Canadian hospitals.

Order sets for COPD exacerbations have been associated with reductions in length of stay [20]. However, there may be unintended consequences of disease-specific order sets. Implementation of order sets for COPD and HF at other Canadian teaching hospitals had marginal gains that were partially offset by shortcomings such as order duplication and variability of order set adoption [21]. Comorbidities can influence the pathophysiologic progression of HF and interfere with drug and diuretic therapy options, disease management, and patient outcomes [22].

Despite order sets being based on clinical practice guidelines resistance to implementation can be present due to practice inertia and lack of generalizability across practices [23]. Optimizing therapy for one condition may result in worsening of another condition [24], necessitating a more holistic approach to care (for instance, in treating acute cardiac decompensation it may be necessary to tolerate a short term worsening of renal function), and that the relative priorities of treatment may evolve over time [25] (for instance, anti-coagulants may be indicated as ideal therapy but cannot be given until renal function improves). 

Our results define a population of patients to prioritize in efforts to reduce total hospital utilization for all patients with HF or COPD based on the number of comorbidities. We have shown that patients admitted to Calgary hospitals with COPD or HF have a similar comorbidity profile to those in other studies. Our results suggest that co-management of the commonly occurring comorbidities in this population may be beneficial in reducing overall hospital utilization, compared with disease-specific order sets.

Limitations

Inherent in the use of administrative data is the risk for misclassification bias. We used validated codes for HF [14] and COPD [13] as well as a validated comorbidity index [26]. If misclassification did occur, it would be non-differential and bias results toward the null. An additional limitation is our assumption that each patient-day in hospital is valued in a consistent manner between conditions, patients, providers, and hospitals and constant regardless of length of admission. In reality, it is likely that individuals would attribute value to days in hospital differently depending on length of admission, severity of disease, and personal preferences [27]. Disease factors such as the type of HF or severity of disease affect hospitalization costs [28] and the fact that hospitals are often reimbursed for initial days in hospital at a higher rate than for subsequent days suggests that our assumption may not be true. Though our study was not designed to address the value of a day in hospital, future analyses could incorporate both quantitative factors such as costs as well as qualitative factors like patient quality of life based on time in hospital to better represent these issues. 

Although our analysis shows that comorbidities contribute to total days in hospital, we did not investigate the impact of single-disease order sets on reducing total days in hospital for patients with HF [29] or COPD [30] (including those with comorbidities). We also did not investigate if order sets that include the management of comorbidities reduce total days in hospital. Future studies could investigate both of these issues.

## Conclusions

Total days in hospital for patients with COPD or HF is highly correlated with the number of comorbidities. Patients with COPD or HF and between one to five comorbidities (compared to those with no comorbidities and those with more than five comorbidities) represent the majority of total days in hospital, and the majority of patients. Patients with COPD and HF admitted to Calgary hospitals commonly present with a ‘syndrome’ of related diagnoses that is similar to comorbidities identified in prior research including hypertension, arrhythmias, diabetes, and fluid and electrolyte imbalances. This highlights the importance of focusing on patients with comorbidities in efforts to reduce hospital utilization, and suggests that concurrent management of commonly occurring comorbidities for HF and COPD patients may be necessary to achieve this goal.
